# Heat Diffusion Kernel Algorithm-Based Interpretation of the Disease Intervention Mechanism for DHA

**DOI:** 10.3390/genes11070754

**Published:** 2020-07-07

**Authors:** Yuan Quan, Hong-Yu Zhang, Jiang-Hui Xiong, Rui-Feng Xu, Min Gao

**Affiliations:** 1 Harbin Institute of Technology Shenzhen Graduate School, Shenzhen 518055, China; quanyuan725@163.com (Y.Q.); xuruifeng@hit.edu.cn (R.-F.X.); 2 Lab of Epigenetics and Advanced Health Technology, Space Science and Technology Institute (Shenzhen), Shenzhen 518117, China; xiongjh77@163.com; 3 Hubei Key Laboratory of Agricultural Bioinformatics, College of Informatics, Huazhong Agricultural University, Wuhan 430070, China; zhy630@mail.hzau.edu.cn

**Keywords:** docosahexaenoic acid (DHA), heat diffusion kernel algorithm, complex disease intervention, mechanism interpretation, gene expression profiles

## Abstract

Docosahexaenoic acid (DHA) is effective in the prevention and treatment of cancer, congenital disorders, and various chronic diseases. According to the omnigenic hypothesis, these complex diseases are caused by disordered gene regulatory networks comprising dozens to hundreds of core genes and a mass of peripheral genes. However, conventional research on the disease intervention mechanism of DHA only focused on specific types of genes or pathways instead of examining genes at the network level, resulting in conflicting conclusions. In this study, we used HotNet2, a heat diffusion kernel algorithm, to calculate the gene regulatory networks of connectivity map (cMap)-derived agents (including DHA) based on gene expression profiles, aiming to interpret the disease intervention mechanism of DHA at the network level. As a result, significant gene regulatory networks for DHA and 676 cMap-derived agents were identified respectively. The biological functions of the DHA-regulated gene network provide preliminary insights into the mechanism by which DHA intervenes in disease. In addition, we compared the gene regulatory networks of DHA with those of cMap-derived agents, which allowed us to predict the pharmacological effects and disease intervention mechanism of DHA by analogy with similar agents with clear indications and mechanisms. Some of our analysis results were supported by experimental observations. Therefore, this study makes a significant contribution to research on the disease intervention mechanism of DHA at the regulatory network level, demonstrating the potential application value of this methodology in clarifying the mechanisms about nutrients influencing health.

## 1. Introduction

Docosahexaenoic acid (DHA), a polyunsaturated fatty acid (PUFA) derived from α-linolenic acid, is an important member of the ω-3 PUFAs [1,2]. DHA is an essential structural component of the human brain (including the cerebral cortex) as well as the retina. However, the human body cannot synthesize DHA or its precursor α-linolenic acid (αLNA). Hence, these nutrients must be obtained through dietary intake [3,4,5]. In recent years, DHA has been recognized to play an important role in the prevention and treatment of cancer, congenital disorders, and various chronic diseases, including cardiovascular diseases, nervous system diseases, and mental diseases [6,7]. Due to its practical biological functions, DHA is included in the official dietary recommendations of The Centers for Disease Control and Prevention (CDC) [2]. Additionally, the mechanisms behind the anti-disease effects of DHA have become an intensively researched topic in modern medicine for a decade.

However, analyses in different DHA-related studies have sometimes resulted in opposite conclusions. For example, a meta-analysis that covered 19 ω-3 PUFA clinical trials related to cardiovascular disease showed that increasing the dose of supplemental ω-3 PUFAs (including DHA and eicosapentaenoic acid (EPA)) did not substantially reduce the risk of cardiovascular disease, coronary heart disease, stroke or heart disease [8]. Another meta-analysis involving 70,000 people also showed that, for patients with a history of coronary heart disease, daily intake of ω-3 PUFAs (1 g/day) did not prevent fatal coronary heart disease, nonfatal myocardial infarction or other cardiovascular diseases [9]. In addition, numerous investigations and studies have shown that ω-3 PUFA supplements are not very beneficial for the prevention and treatment of chronic diseases [10,11,12,13,14,15]. In 2010, the National Institutes of Health (NIH) released its State-of-the-Science Conference Statement: preventing Alzheimer’s disease and cognitive decline, which stated that several longitudinal studies have shown long-chain ω-3 PUFAs to be associated with reduced risk of cognitive decline. However, the statement concludes that the current data are insufficient to support the provision of dietary supplement recommendations to prevent cognitive decline [16]. In general, the medical efficacy and action mechanism of DHA remain poorly understood in the fields of biology and medicine.

Recently, a review published in Cell proposed the omnigenic hypothesis, which states that complex diseases are often caused by disordered gene regulatory networks composed of dozens to hundreds of core genes and a mass of peripheral genes [17]. To elucidate the pathogenesis of complex diseases, researchers need to interpret the regulatory associations between various genes and construct the gene regulatory networks. Accordingly, we suspected that DHA may intervene in complex diseases, such as cancer, cardiovascular diseases, and nervous system diseases, based on gene regulatory networks. However, most of the current research on DHA focuses only on specific types of genes or specific pathways, and there is a lack of research to elucidate the disease intervention mechanism of DHA at the regulatory network level. We suggested that this is an important reason for the conflicting results derived from different DHA-focused studies.

HotNet diffusion-oriented subnetworks (HotNet2) is a representative heat diffusion kernel algorithm [18]. This algorithm not only considers the heat of genes (reflecting their biological importance) but also integrates the topology of protein-protein interactions (PPIs). Based on HotNet2, Leiserson et al. used the somatic mutation data of 12 different cancer types in The Cancer Genome Atlas (TCGA) project to map the DNA mutation frequency of cancer patients as gene heats; in that manner, they have identified gene regulatory networks significantly associated with 12 types of cancers [18]. These HotNet2-identified networks included several common cancer pathways, such as the TP53, PI3K, NOTCH, and RTK signaling pathways, confirming the high value of the HotNet2 algorithm in constructing gene regulatory networks and interpreting disease mechanisms. In addition, researchers have compared HotNet2 to HotNet and to two standard methods of pathway enrichment (i.e., DAVID and Gene Enrichment Analysis (GSEA)). They proved that HotNet2 can identify more accurate pathways or gene networks related to diseases than these standard methods [18].

**Figure 1:**
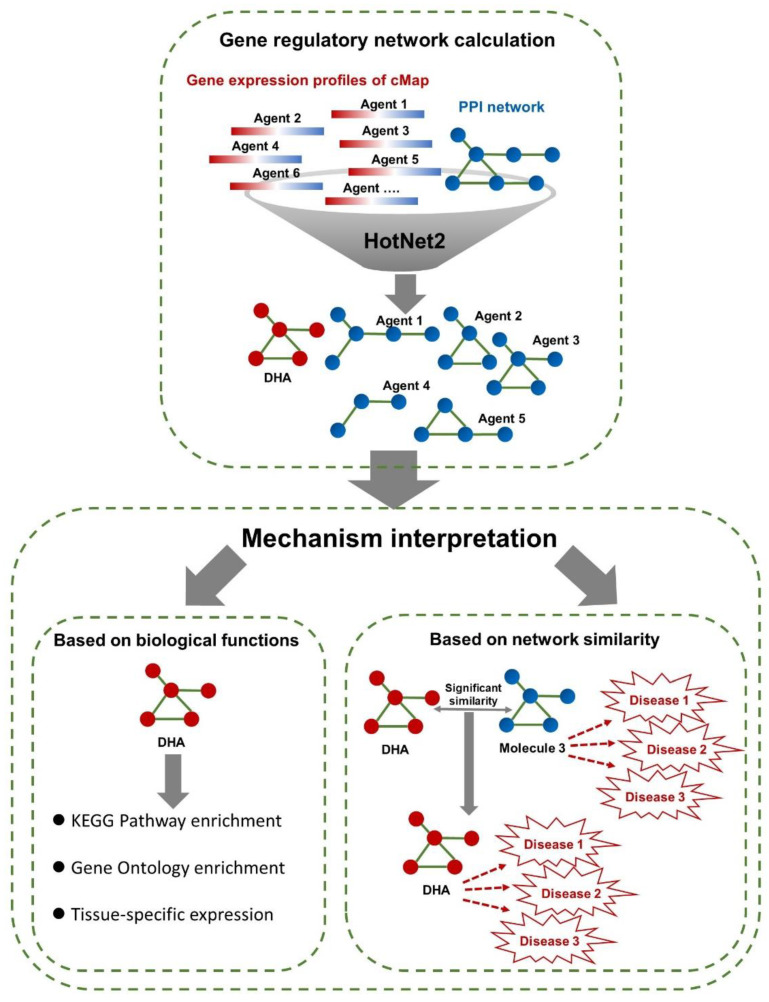
Data processing pipeline of HotNet2-based gene regulatory network construction and mechanism interpretation. In this study, the gene expression profiles of cMap (including docosahexaenoic acid (DHA)) were downloaded from the cMap database (https://www.broadinstitute.org/cmap/) [19]. The PPI network was obtained from HINT, iRefIndex and Multinet [18]. For each cMap-derived agent, during the calculation of gene regulatory networks by HotNet2, the expression values of probes were used as initial heat vectors for the corresponding genes. The default parameters and procedures of the HotNet2 algorithm (https://github.com/raphael-group/hotnet2) were applied in our study [18]. Then, we interpreted the disease intervention mechanism of DNA based on enriched biological functions for regulatory network-containing genes, involving KEGG pathways, GO molecular functions, and tissue-specific expression. In addition, we compared the gene regulatory networks of DHA with those of cMap-derived agents, which allowed us to predict the pharmacological effects and disease intervention mechanism of DHA by analogy with similar agents with clear indications and mechanisms.

In this study, we first performed calculations with HotNet2 to construct the gene regulatory networks of connectivity map (cMap)-derived agents (including DHA) based on their gene expression profiles and PPIs (Figure 1). Then, we interpreted the disease intervention mechanism of DHA by performing biological functional analyses for the regulatory network-containing genes (Figure 1). Next, by comparing the similarities of the gene regulatory networks of DHA and those of other cMap-derived agents, we identified some agents similar to DHA (Figure 1). Because some of these similar agents have clear indications and treatment mechanisms, we can predict the potential target diseases and intervention mechanisms of DHA by analogy.

## 2. Data Sources and Methods

### 2.1. cMap Data Preprocessing

The gene expression profiles of cMap were downloaded from the cMap database (https://www.broadinstitute.org/cmap/) [19]. At present, the cMap dataset contains ~6100 expression profiles related to 5 cultured human cell lines (i.e., HL60, PC3, MCF7, ssMCF7, and SKMEL5) treated with 1309 agents at multiple time points, and ~900 control profiles are included as well [19].

The raw data of gene expression profiles were first normalized by Robust Multi-array Average expression measure according to Xiong et al.’s processing pipeline [20]. When the test group and the control group for the same agent had several repeated experiments, we calculated the average value of the repeated data of the test group and named it *t*; we also calculated the average value of the repeated data of the control group and named it *c*. Then, we used the following formula to merge the test groups and the control groups of the agents:

(1) $$a=\frac{t-c}{\left(t+c\right)/2}$$

where *a* is the expression value of the probe after treatment with the agent in the human cell line, *t* is the average probe value of the repetitive test group, and *c* is the average probe value of the repetitive control group.

For each cMap-derived agent, the expression values of probes were usually determined under different conditions. Therefore, the medians of the probe expression values were used to represent the expression profile. After data processing, 1309 columns of gene expression profiles were obtained in this study, corresponding to 1309 cMap-derived agents.

### 2.2. Gene Regulatory Network Calculation

In this study, we applied the HotNet2 algorithm to calculate the gene regulatory networks of 1309 cMap-derived agents. The algorithm requires two types of data input: initial heat vectors of genes and PPI information [18]. For each cMap-derived agent, during the calculation of gene regulatory networks by HotNet2, the expression values of probes were used as initial heat vectors for the corresponding genes. When a gene corresponded to multiple probes, the maximum expression value was used as the initial heat vector.

Considering the computational efficiency of HotNet2 (the number of gene inputs cannot be too large), we collected only the 1000 genes with the most upregulated expression and the 1000 genes with the most downregulated expression for HotNet2 calculation. The PPI network was obtained from HINT, iRefIndex, and Multinet [18]. In the process of HotNet2 calculation, two parameters need to be involved: β and δ. The value of β is selected from the PPIs, independently of any initial heat vectors of genes. The value of δ is chosen such that large connected components are not found using the observed distribution of gene initial heat on random networks with the same degree distribution as the observed network. And HotNet2 can select values for β and δ using automated procedures [18].

### 2.3. Biological Function Enrichment

We interpreted the disease intervention mechanism of DHA based on biological functions, including Kyoto Encyclopedia of Genes and Genomes (KEGG) pathway enrichment, Gene Ontology (GO) molecular function enrichment, and tissue-specific expression analysis. First, the KEGG pathways and GO molecular functions of genes included in the DHA gene regulatory network were enriched using the records in Database for Annotation, Visualization, and Integrated Discovery (DAVID, https://david.ncifcrf.gov/) [21]. The corresponding disease catalogs of the KEGG pathways were downloaded from the KEGG Pathway Database (https://www.genome.jp/kegg/pathway.html) [22].

We performed human tissue-specific expression analysis for the regulatory network-containing genes of DHA by using the Enrichr database (https://amp.pharm.mssm.edu/Enrichr/) [23]. Considering the quality and credibility of the tissue-specific gene expression data, this study selected only the Genotype-Tissue Expression (GTEx) project as a data source.

### 2.4. Similarity Calculation of Gene Regulatory Networks

In this study, the hypergeometric test was used to calculate the similarity of HotNet2-calculated gene regulatory networks between DHA and other cMap-derived agents. The calculation formula is as follows:

(2) $$p-value=1-F\left(x-1/M,K,N\right)=1-\overset{x-1}{\underset{i=0}{\sum }}\frac{\left(\begin{array}{c} K\\ i \end{array}\right)\left(\begin{array}{c} M-K\\ N-i \end{array}\right)}{\left(\begin{array}{c} M\\ N \end{array}\right)}$$

where *x* is the number of genes in the intersection of regulatory networks between DHA and a cMap-derived agent, *M* is the number of genes in the union of regulatory networks between DHA and a cMap-derived agent, *K* is the number of genes in DHA’s regulatory network, and *N* is the number of genes in a cMap-derived agent’s regulatory network. Then, we used the *p*-value calculated by this test to measure the similarity of two gene regulatory networks. In this study, we used a commonly accepted *p*-value of 0.05 as the threshold of significant similarity. When the *p*-value of hypergeometric test is less than or equal to 0.05, we think these two gene regulatory networks are significantly similar. The smaller the *p*-value, the more similar the two networks are.

## 3. Results

### 3.1. Gene Regulatory Networks of cMap Agents

**Figure 2:**
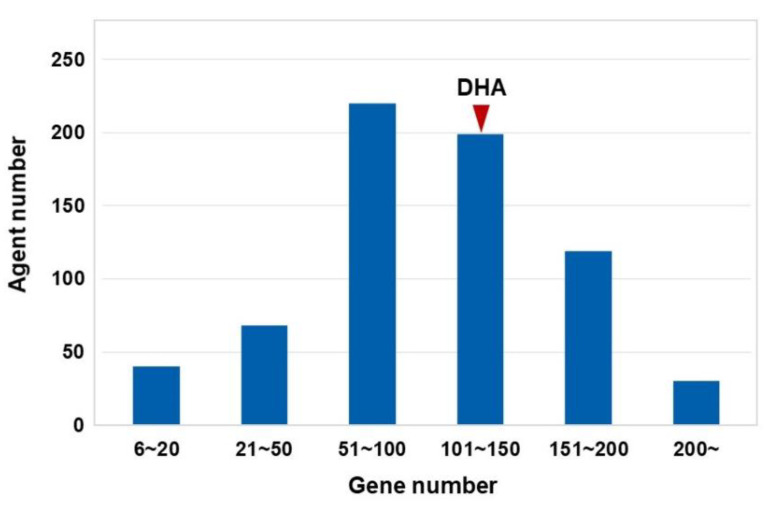
Distribution patterns of numbers of genes in HotNet2-calculated regulatory networks. The regulatory network of DHA contains 104 genes.

In this study, we used the HotNet2 algorithm to construct gene regulatory networks for DHA and other cMap agents (Figure 1). During the HotNet2 calculation, the expression values of probes were used as initial heat vectors for the corresponding genes. As a result, significant gene networks for 677 cMap-derived agents, including DHA, were successfully identified from gene expression profiles (Table S1). Regarding the distribution pattern of the numbers of genes in these regulatory networks, more than 50% of the networks contained 50–150 proteins (Figure 2). Among these networks, the gene regulatory network related to DHA contains 104 genes (Table S1). Notably, the numbers of genes in these significant networks are consistent with the numbers of core genes in complex disease-regulated networks according to the omnigenic hypothesis. Therefore, we interpreted the disease intervention mechanism of DHA based on these HotNet2-calculated regulatory networks.

### 3.2. Interpretation of Disease Intervention Mechanisms Based on Biological Functions

#### 3.2.1. Interpretation by KEGG Pathway Enrichment

This research elucidated the anti-disease mechanism of DHA by analyzing the biological function of the DHA-regulated gene network. First, the biological functions of the KEGG pathways and GO molecular functions were enriched for 104 genes included in the network, as determined by the DAVID tool. Thus, the DHA-regulated network-containing genes were significantly enriched in 29 KEGG pathways and 52 GO molecular function terms (*p*-value ≤ 0.05) (Tables S2 and S3).

**Figure 3:**
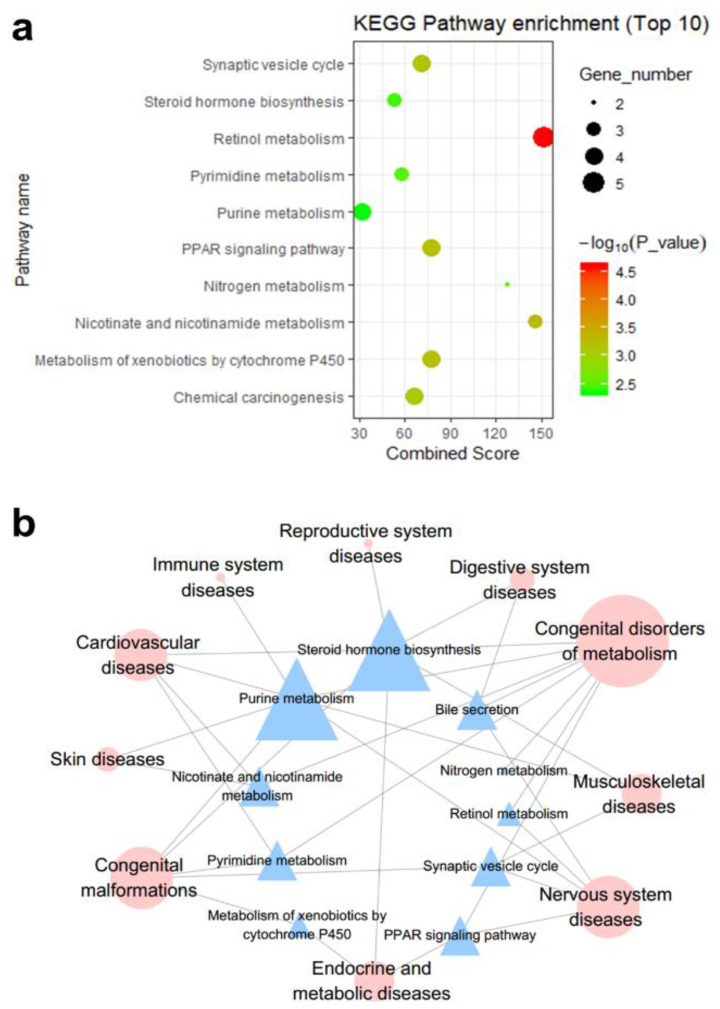
(**a**) The enriched KEGG pathways of DHA regulatory network-containing genes (top 10). The KEGG pathways of genes included in the DHA gene regulatory network were enriched using the records in Database for Annotation, Visualization, and Integrated Discovery (DAVID, https://david.ncifcrf.gov/) [21]. (**b**) The correlations between KEGG pathways and disease classes. The blue triangles represent the KEGG pathways, and the red circles represent the disease classes. The corresponding disease catalogs of the KEGG pathways were downloaded from the KEGG Pathway Database (https://www.genome.jp/kegg/pathway.html) [22].

As shown in Figure 3b, according to the disease association annotations with the pathways in the KEGG pathway database, the top 10 significant KEGG pathways enriched with genes in the DHA-regulated network are associated with different disease classes (Figure 3). For example, eight pathways are related to the disease class “congenital disorders of metabolism”, which is the disease class that involves the most pathways. The disease class “congenital malformations” has the second most pathways, with five. Consistent with our predictions, a study conducted by Manley et al. on fish oil supplementation in infants showed that for infants born at <33 weeks of gestation, DHA supplementation can reduce the incidence of bronchopulmonary dysplasia in boys and in all infants weighing <1250 g [24]. In addition, a random double-blind study conducted by Helland et al. showed that supplementation of ω-3 PUFA during pregnancy and lactation may be beneficial to the intellectual development of the children at 4 years of age [25]; in addition, Lauritzen et al. found that maternal supplementation with fish oil during the first four months of lactation can positively affect the intellectual development of female children [26]. Dunstan et al. found that children whose mothers received supplementation with high levels of DHA during pregnancy had significantly better hand-eye coordination than children in the placebo group [27]. Therefore, the above pathways deserve further attention when studying the congenital disease intervention mechanism of DHA.

In addition, it is well known that nervous system protection and treatment of cardiovascular diseases are the most common functions of DHA; hence, the pathways associated with these two disease classes are worthy of attention. Five pathways, namely, purine metabolism, bile secretion, retinol metabolism, the peroxisome proliferator-activated receptor (PPAR) signaling pathway, and the synaptic vesicle cycle, are related to nervous system diseases (Figure 3b). Many studies have proven the associations between DHA and nervous system regulation. Lee et al. showed that fish oil supplementation potentially improves the cognitive function of elderly individuals with mild cognitive impairment (MCI) [28], and Heras-Sandoval et al. reported that DHA improves the cognition of patients with early-stage Alzheimer’s disease by regulating the activity of glial cells [29]. Stonehouse et al. found that DHA supplementation can improve the memory and memory reaction time of healthy young adults with low DHA intake through their habitual diet [30]. In addition, we know that neurological diseases are closely related to mental diseases, and many studies have shown that DHA does indeed have the potential to treat mental diseases. Levant et al. used female Long-Evans rats proved that the decrease of DHA tissue level may be one of the causes of postpartum depression [31]. Jiang et al. found that DHA has antidepressant effects, including various effects on the monoamine neurotransmitter system, red blood cell membranes, and HPA axis [32].

Additionally, four pathways are associated with cardiovascular diseases: pyrimidine metabolism, steroid hormone biosynthesis, purine metabolism, and nicotinate and nicotinamide metabolism (Figure 3b). The interventions with cardiovascular diseases are other important effects of DHA. McManus et al. demonstrated that a single supplementary dose of DHA can significantly improve postprandial arterial stiffness [33], and epidemiological studies have shown that high-dose DHA intake is associated with reduced rates of myocardial infarction, atherosclerosis, and other ischemic pathologies [34]. Arai et al. found that fish oil with a high DHA content can suppress weight gain by inhibiting lipid synthesis in female KK mice and shows an anti-obesity effect [35]. In summary, we proposed that the above KEGG pathways deserve further study in relation to the disease intervention mechanism of DHA.

#### 3.2.2. Interpretation by GO Molecular Functional Enrichment

**Figure 4:**
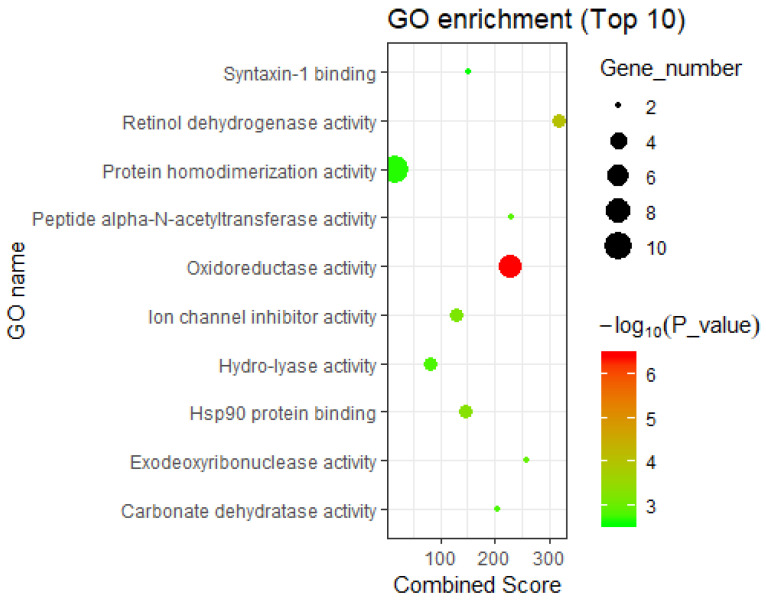
The enriched GO molecular functions of DHA regulatory network-containing genes (top 10). The GO molecular functions of genes included in the DHA gene regulatory network were enriched using the records in Database for Annotation, Visualization, and Integrated Discovery (DAVID, https://david.ncifcrf.gov/) [21].

On the other hand, GO molecular functional enrichment analysis of the DHA-regulated network (Table S3), showed that the network-involved genes were significantly related to the GO molecular function “oxidoreductase activity, acting on the CH-OH group of donors, NAD or NADP as acceptor” (*p*-value = 3.77 × 10^−7^) (Figure 4), involving the genes *UGDH, BDH1, EHHADH, ADH1A, ME1, RDH5,* and *DHRS3*. Interestingly, Newell et al. reviewed the relationship between DHA-rich fish and the reduced incidence of certain cancers (especially colorectal cancer, prostate cancer, and breast cancer), because DHA can enhance many cellular processes, including regulation of the oxidative stress response and activation of PPAR [36]. Therefore, this GO molecular function may be an approach by which DHA can be used in the treatment of cancer. 

In addition, these DHA-regulated genes are significantly related to the GO molecular function “retinol dehydrogenase activity” (*p*-value = 8.81 × 10^−5^) (Figure 4). Ma et al.’s study showed that mutation of retinol dehydrogenase is associated with nonalcoholic fatty liver disease, and retinol dehydrogenase plays a role in nonalcoholic fatty liver disease through its enzymatic activity [37]. Therefore, genes in the functional category “retinol dehydrogenase activity” (i.e., *ADH1A*, *RDH5*, and *DHRS3*) are worthy of attention in the study of the anti-cardiovascular disease effects of DHA.

Third, the DHA-regulated genes were significantly enriched in the “Hsp90 protein binding” GO molecular function (*p*-value = 5.01 × 10^−4^) (Figure 4), involving *CHORDC1*, *USP19*, and *FKBP6*. Members of the heat shock protein 90 (Hsp90) family can participate in regulating several important biological functions in humans. For example, tumor necrosis factor receptor-associated protein 1 (*TRAP1*), a major member of the Hsp90 family, is an important molecular chaperone. Studies have shown that regulation of TRAP1 activity can effectively prevent cardiomyocyte damage caused by hypoxia, maintain the vitality of myocardial cells and the mitochondrial membrane potential, and protect myocardial cells, thereby inhibiting cardiovascular diseases [38]. In addition, *TRAP1* can also protect astrocytes from ischemic damage, which plays a direct role in regulating human learning and memory [36]. Moreover, numerous recent studies have shown that the abnormal expression of *TRAP1* is closely related to the occurrence and development of various tumors [38]. The diseases mentioned above are common diseases affected by DHA intervention. In summary, we can gain preliminary insights into the disease intervention mechanism of DHA according to the biological functions of genes in the HotNet2-calculated gene regulatory network.

#### 3.2.3. Interpretation by Tissue-Specific Expression Analysis

**Figure 5:**
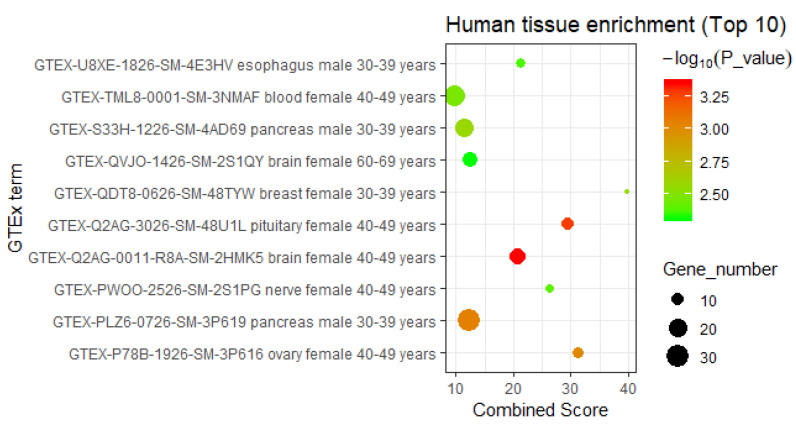
The enriched human tissues of DHA regulatory network-containing genes (top 10). The human tissues of genes included in the DHA gene regulatory network were enriched using the records in Enrichr database (https://amp.pharm.mssm.edu/Enrichr/) [23]. Considering the quality and credibility of the tissue-specific gene expression data, this study selected only the Genotype-Tissue Expression (GTEx) project as a data source.

Then, we carried out human tissue-specific expression analysis for the DHA regulatory network-containing genes via the Enrichr database [23] (Table S4). We found that these regulatory network-contained genes were most significantly enriched in the gene sets specifically expressed in the brains of 40- to 49-year-old and 60- to 69-year-old females (*p*-value = 4.45 × 10^−4^ and *p*-value = 4.93 × 10^−3^, respectively) (Figure 5). In addition to being specific for the female brain, these genes are also significantly associated with gene sets that are specifically expressed in other human nervous system tissues in female individuals, including the pituitary (40–49 years, *p*-value = 5.24 × 10^−4^) and nerves (40–49 years, *p*-value = 4.10 × 10^−3^) (Figure 5). These results are consistent with the extensively reported biological function of DHA, namely, regulating the human nervous system. In addition, the tissue-specific expression analysis also implies that the intervention effects of DHA in the human body depend on gender and age, and we inferred that DHA is more effective in treating neurological disease in 40- to 49-year-old females than in other individuals.

### 3.3. Interpretation of the Disease Intervention Mechanism Based on Network Similarity

To interpret the disease intervention mechanism of DHA, we calculated the similarities of gene regulatory networks between DHA and 676 other cMap-derived agents by the hypergeometric test and used the *p*-value to represent the degrees of similarity between networks. According to the comprehensive drug database SCG-Drug (http://zhanglab.hzau.edu.cn/scgdrug) [39], which has integrated information on agent indications from multiple sources and standardized the indication descriptions of agents using MetaMap [39], there are 320 of 676 cMap-derived agents with clear indication annotations (Table S5). As shown in Table 1, based on the *p*-value of the hypergeometric test for gene regulatory networks, the top 20 cMap-derived agents (i.e., propafenone, clopamide, fenbufen, etanidazole, thiethylperazine, phenelzine, zalcitabine, dydrogesterone, leflunomide, albendazole, iloprost, alclometasone, lomustine, rescinnamine, benzocaine, proguanil, meclocycline, diethylstilbestrol, midecamycin, pyrvinium) that are most similar to DHA were selected for predicting the potential efficacy and disease intervention mechanism of DHA.

**Figure 6:**
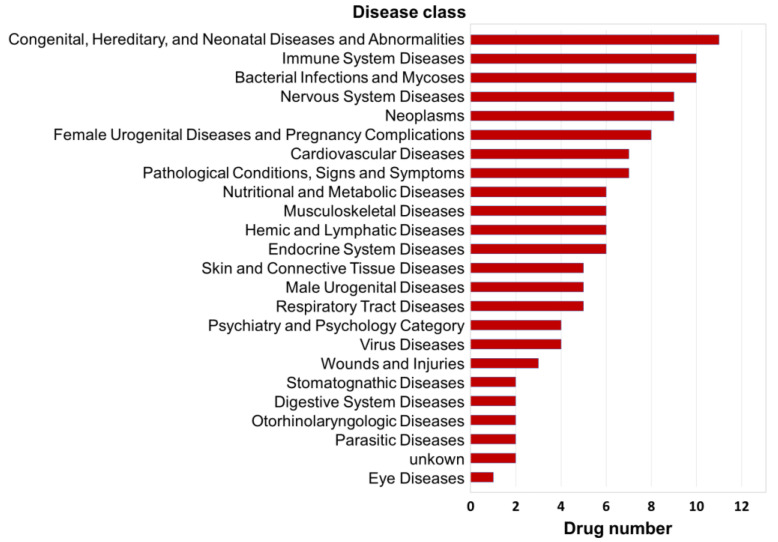
Distribution patterns of the disease classes of the top 20 agents similar to DHA. The indications of agents were downloaded from the comprehensive drug database SCG-Drug (http://zhanglab.hzau.edu.cn/scgdrug) [39]. Additionally, according to the NCBI Medical Subject Headings (MeSH) database (https://www.ncbi.nlm.nih.gov/mesh/), the standardized disease descriptions of agent indications can be divided into 24 disease classes.

Additionally, according to the NCBI Medical Subject Headings (MeSH) database (https://www.ncbi.nlm.nih.gov/mesh/), the standardized disease descriptions of agent indications can be divided into 24 disease classes. Through the integrated analysis of disease classes for the indications of the 20 similar agents listed above, we found that they were most strongly associated with the disease class of “congenital, hereditary, and neonatal diseases and abnormalities”, which involves 11 agents. Second, there were 10 agents each related to the disease classes of “bacterial infections and mycoses” and “immune system diseases”; third, there were 9 drugs each related to the disease classes of “neoplasms” and “nervous system diseases” (Figure 6). Accordingly, we inferred that prevention of congenital diseases, prevention of neoplasms, immune system protection, and neurological regulation are the most fundamental biological effects of DHA, which is consistent with the findings of previous studies.

In addition, notably, 7 agents (alclometasone, clopamide, iloprost, leflunomide, propafenone, rescinnamine, and zalcitabine) with gene regulatory networks similar to that of DHA were related to cardiovascular disease (Figure 6), which is one of the most important medical functions of DHA. Therefore, we speculate that these 7 agents with clear treatment mechanisms can be used to analyze the disease intervention mechanism of DHA by analogy in the future.

## 4. Conclusions

It is well known that DHA is an essential ω-3 PUFA for brain nutrition, supporting nerve conduction and synaptic growth in the brain. Thus, effective intake of DHA can prevent the occurrence of certain congenital diseases. In addition, DHA can prevent the deposition of cholesterol on the blood vessel walls, which can effectively prevent or reduce the occurrence of atherosclerosis and coronary heart disease. In recent years, studies have found that DHA is effective as an adjuvant for cancer treatment. In general, due to its extensive efficacy for disease intervention, DHA has attracted considerable attention from the biological and medical community. However, the disease intervention mechanism of DHA is still unclear.

According to the omnigenic hypothesis, complex diseases are caused by disordered gene regulatory networks composed of dozens to hundreds of core genes and a mass of peripheral genes. Accordingly, we suggested that DHA may intervene in diseases through a gene regulatory network. In this study, based on cMap database-obtained gene expression profiles, we used HotNet2 to calculate the gene regulatory networks and identified significant gene regulatory networks for DHA and 676 additional cMap-derived agents. First, we interpreted the disease intervention mechanism of DNA based on enriched biological functions for DHA regulatory network-containing genes, involving KEGG pathways, GO molecular functions, and tissue-specific expressions. Next, we compared the gene regulatory networks between DHA and other cMap-derived agents. Through the integrated analysis of disease classes for the agents similar to DHA, we found that they had the strongest associations with the disease classes of “congenital, hereditary, and neonatal diseases and abnormalities”, “neoplasms”, “nervous system diseases”, and “cardiovascular disease”, which are consistent with the widely reported medical efficacy of DHA. Some of our analysis results were supported by experimental observations.

In conclusion, because this study analyzed the disease intervention mechanism of DHA at the level of gene regulatory networks rather than a single biological element, it is expected to overcome the limitations of traditional research. Furthermore, because the input required by the HotNet2 algorithm, i.e., the initial heat of genes and the PPI data, are becoming more widely available in the biological field, our data processing pipeline can be readily extended to mechanism interpretation for other nutrients. However, this research still has some limitations. First, the effectiveness of the HotNet2-identified networks is extremely dependent on the quality of the initial input heat of genes. Therefore, before calculating the network, the credibility of the associations between genes and phenotypes need to be verification by researchers. Second, we only used the broad-spectrum PPIs in this study. However, it is well known that different nutrients can affect different organs or tissues of the human body, thereby producing different expression profiles. Therefore, if the organ- or tissue-specific PPIs can be used as input based on a priori knowledge, then a network that is more in line with biological reality may be identified. Fortunately, we believe that with the accumulation of biological data, the above limitations will be alleviated. Additionally, it is worth noting that, in addition to DHA, cMap also has accumulated gene expression profiles of other agents whose medical efficacy and treatment mechanisms have not been fully resolved. Therefore, we expect that our methodology can be applied to future agent-focused research.

## Figures and Tables

**Table 1:** Predicted cMap-derived agents similar to DHA (top 20).

Agent	*p*-Value ^a^	Agent	*p*-Value ^a^
Propafenone	3.59 × 10^−6^	Iloprost	2.08 × 10^−4^
Clopamide	6.47 × 10^−6^	Alclometasone	2.56 × 10^−4^
Fenbufen	4.89 × 10^−5^	Lomustine	3.40 × 10^−4^
Etanidazole	1.00 × 10^−4^	Rescinnamine	3.51 × 10^−4^
Thiethylperazine	1.22 × 10^−4^	Benzocaine	4.70 × 10^−4^
Phenelzine	1.25 × 10^−4^	Proguanil	5.11 × 10^−4^
Zalcitabine	1.32 × 10^−4^	Meclocycline	5.61 × 10^−4^
Dydrogesterone	1.62 × 10^−4^	Diethylstilbestrol	7.35 × 10^−4^
Leflunomide	1.64 × 10^−4^	Midecamycin	9.59 × 10^−4^
Albendazole	1.85 × 10^−4^	Pyrvinium	1.07 × 10^−3^

^a^ Calculated by hypergeometric test.

## Supplementary Materials

The following are available online at https://www.mdpi.com/2073-4425/11/7/754/s1. Table S1: Significant subnetworks for 677 cMap-derived agents calculated by HotNet2; Tale S2: The enriched KEGG pathways of DHA regulatory network-containing genes; Table S3: The enriched GO molecular functions of DHA regulatory network-containing genes; Table S4: The enriched human tissues of DHA regulatory network-containing genes; Table S5: The basic informations of similar cMap-derived agents to DHA.
